# Socio-Economic and Health Access Determinants of Breast and Cervical Cancer Screening in Low-Income Countries: Analysis of the World Health Survey

**DOI:** 10.1371/journal.pone.0048834

**Published:** 2012-11-14

**Authors:** Tomi F. Akinyemiju

**Affiliations:** Department of Epidemiology, University of Michigan School of Public Health, Ann Arbor, Michigan, United States of America; Kenya Medical Research Institute - Wellcome Trust Research Programme, Kenya

## Abstract

**Background:**

Breast and Cervical cancer are the two most common cancers among women in developing countries. Regular screening is the most effective way of ensuring that these cancers are detected at early stages; however few studies have assessed factors that predict cancer screening in developing countries.

**Purpose:**

To assess the influence of household socio-economic status (SES), healthcare access and country level characteristics on breast and cervical cancer screening among women in developing countries.

**Methods:**

Women ages 18–69 years (cervical cancer screening) and 40–69 years (breast cancer screening) from 15 developing countries who participated in the 2003 World Health Survey provided data for this study. Household SES and healthcare access was assessed based on self-reported survey responses. SAS survey procedures (SAS, Version 9.2) were used to assess determinants of breast and cervical cancer screening in separate models.

**Results:**

4.1% of women ages 18–69 years had received cervical cancer screening in the past three years, while only 2.2% of women ages 40–69 years had received breast cancer screening in the past 5 years in developing countries. Cancer screening rates varied by country; cervical cancer screening ranged from 1.1% in Bangladesh to 57.6% in Congo and breast cancer screening ranged from 0% in Mali to 26% in Congo. Significant determinants of cancer screening were household SES, rural residence, country health expenditure (as a percent of GDP) as well as healthcare access.

**Discussion:**

A lot more needs to be done to improve screening rates for breast and cervical cancer in developing countries, such as increasing health expenditure (especially in rural areas), applying the increased funds towards the provision of more, better educated health providers as well as improved infrastructure.

## Introduction

Cancer incidence and mortality rates have been declining in developed, western nations due mainly to a reduction in risk factors such as smoking and improved screening and treatment regimens [1]–[5]. However, the opposite trend is being observed in low-income countries; cancer incidence and mortality rates have been increasing and are projected to increase at even faster rates [6]–[10]. Of the 12 million incident cases and 8 million deaths due to cancer worldwide in 2008, 53% of the new cases and 65% of the deaths occurred in less developed countries [11]. There are several suggested reasons for these increases including the rising popularity of western lifestyle that includes smoking, lower physical activity levels, lower reproduction rates and higher calorie intake [7], [12]–[16]. In addition, developing countries are disproportionately affected by infectious agents that may cause cancer such as the Human Papillomavirus (for cervical cancer), H. Pylori (for stomach cancer) and Hepatitis B and C (for liver cancer) [1], [17].

The high cancer mortality rates can also be partly attributed to lack of adequate health infrastructure and healthcare personnel in developing counties [18]–[21]. One major approach to reducing mortality rates involves improving access to cancer screening. Breast and cervical cancer are the two most common cancers in most low-income countries, accounting for 19% and 23% of all cancers respectively [22]–[24]. Fortunately, adequate screening is capable of identifying these cancers at early stages where treatment regimens are effective, uncomplicated and cheaper [25]. Unfortunately, cancer screening rates are very low in developing countries; only 19% of women were screened for cervical cancer in developing countries, compared with over 60% in developed countries [26]. Also, less than 1% of women ages 25 to 64 years in Bangladesh had received a Pap test in the past 3 years [27]. In addition to lack of infrastructure and personnel, competing healthcare crises in poor countries due to HIV/AIDS or infant mortality may further complicate the receipt of adequate cancer screening [11]. It is therefore very important to understand the factors that affect cancer screening in order to develop programs to make it easier for women to get screened in both rural and urban areas.

Two recent studies have examined the impact of income inequality on cervical cancer screening among countries that participated in the WHS [26], [27]. These studies contribute to better understanding of the factors that impact screening. The purpose of this study is to further explore determinants of breast and cervical cancer screening among women, focusing on those residing in low-income countries. Very little has been published so far about the factors that affect cancer screening in low income countries, and this may be due to the lack of quality survey data on cancer screening. However, the World Health Survey provides a unique opportunity to use high quality data collected in a standardized format to examine specific factors such as SES and access to healthcare that influence adequate breast and cervical cancer screening. In addition, country level characteristics that reflect the strength and quality of the public health infrastructure such as health expenditure and under-5 mortality rates may also predict screening and were assessed in this study. We hypothesize that low socio-economic status and poor access to healthcare are major predictors of cancer screening in low-income countries.

## Methods

### Data Sources and Analytic Samples

This study utilized data from the 2002–2003 World Health Survey (WHS) conducted in 70 countries by the World Health Organization. The details of the survey have been published elsewhere [27], [28] and is available online at http://www.who.int/healthinfo/survey/en/. In brief, the WHS is a cross-sectional, multistage cluster survey that utilizes a probability sampling design in which every individual in the sampling frame has a known and non-zero probability of being in the survey sample. Data from each country was designed to be nationally representative of the country's eligible population at the time of the survey. Eligible survey participants were men and women ages 18 years and older who were surveyed through private (where possible), face-to-face interviews by survey interviewers [29]. The survey captured information on demographics, health status, risk factors, health system responsiveness, health expenditure and coverage, access and utilization of health services. Information was collected at the household and individual level. Survey materials were translated into local languages where appropriate and reviewed according to standard WHO protocol.

This analysis was restricted to WHS data from low income countries. Low-income was defined according to the World Bank's classification of countries based on the 2010 Gross National Income (GNI) per capita. Based on this criterion, low-income countries are those that have a GNI per capita of $1,005 or less. Details of this categorization can be found at http://data.worldbank.org/about/country-classifications. In addition, three other country level characteristics were obtained from the World Bank; total health expenditure as a percentage of GDP, under-5 mortality rate per 1,000 and gross national income per capita.

Data from 15 low-income countries that participated in the WHS were used in this analysis. The countries included are: Chad, Mali, Congo (Brazzaville), Comoros, Laos, Zimbabwe, Burkina Faso, Nepal, Mauritania, Myanmar, Ghana, Kenya, Malawi, Ethiopia and Bangladesh. Data from Zambia was not included in the analysis because only one cluster represented the entire country which did not permit for analysis using valid survey statistical methods. In addition, data from 2 strata in Nepal and Kenya were dropped from the variance estimation process due to missing data on demographic information. Survey sampling analytic procedures was employed to account for clustering and unequal probability of selection. The analytic dataset was restricted to women ages 18 to 69 (30,509 women) for cervical cancer screening and 40 to 69 (10,860 women) for breast cancer screening. Questions about health care access were only asked of respondents who had visited a healthcare facility for themselves or their children in the past 12 months. Therefore, this analysis focused on women in the specified age groups who had been to a healthcare facility in the past 12 months. There were 10,021 women ages 18 to 69 and 4,009 women ages 40–69 that fulfilled the study criteria.

### Data Management

#### Cancer Screening

There were three questions in the WHS addressing cancer screening, two about cervical cancer screening asked of women ages 18 to 69 years, and one about mammography use asked of women ages 40 to 69 years. 1. “When was the last time you had a pelvic examination, if ever? (By pelvic examination, I mean when a doctor or nurse examined your vagina and uterus?)”. 2. “The last time you had the pelvic examination, did you have a PAP smear test? (By PAP smear test, I mean did a doctor or nurse use a swab or stick to wipe from inside your vagina, take a sample and send it to a laboratory?)”. 3. “When was the last time you had a mammography, if ever? (That is, an xray of your breasts taken to detect breast cancer at an early stage)”. For this analysis, cervical cancer screening was defined as a pelvic examination with or without a pap test in the past 3 years among women ages 18–69 years; while breast cancer screening was defined as a mammography test in the past 5 years among women ages 40–69 years.

#### Socio-Demographic Variables

Marital status was categorized as single, married/cohabiting or divorced/separated/widowed. Education level was categorized as primary school or less, secondary school or college and higher. Employment was categorized as government employee, self-employed or not working for pay. Age was categorized as <40 years, 40–60 years and >60 years. Residence was categorized as rural or urban. All variables are based on self-reports.

#### Socio-Economic Status

Permanent income indicators for low-income countries were used to define socio-economic status for survey respondents. Survey questions assessed ownership of a range of assets from chairs, tables, ploughs and buckets to cars, mobile phones, washing machines and refrigerators. Country-specific items were also added to the list of permanent income indicators to account for differences between countries. To create a composite measure of socio-economic status, country-specific principal components analysis (PCA) was performed using these permanent income indicators. This approach has been used in previous studies using data from low-income countries where income and education variables are often inaccurate and not likely to capture the full extent of an individual's socio-economic status [30]–[33]. In brief, an SES score was calculated for each household by weighting each income indicator by the coefficient of the first principal component. All individuals present in the household were assigned the same household SES score, and the score was categorized into tertiles; poorest, middle and richest.

#### Access to Healthcare

Individual access to healthcare was assessed in this study using questions related to health system responsiveness in the WHS. There were four variables used to define access to healthcare: 1. Last place visited in the past 12 months (e.g. government facility, private facility, NGO, or other); 2. Last healthcare provider visited (e.g. medical doctor, nurse, midwife, traditional practitioner, etc); 3. How long it took to get there (in minutes); 4. Mode of transportation (e.g. private car, public transportation, biking or walking).

#### Country Covariates

Country level healthcare infrastructure characteristics were assessed using three variables obtained from the World Bank; health expenditure as a percent of GDP, under-5 mortality, and the Gross National Income. Health expenditure as a percent of GDP is a measure of public and private health expenditure on preventive and curative health services. Under-five mortality rate represents the probability per 1,000 that an infant will die before age five if current age-specific mortality rates apply. GNI per capita is a measure of the total value of all products and services produced by the domestic economy of a country divided by the population of the country, measured in US dollars.

### Statistical Analysis

SAS statistical software (SAS, Version 9.2) was used for data analysis. Survey analytic procedures were used to account for the complex survey design. Sample weights that represent the population of each specific country, and stratum codes that are nested within each country based on the country's survey design were incorporated into the analysis. Descriptive statistics were generated using chi-square tests with the SURVEYFREQ procedure in SAS. The outcome variables were dichotomized into recent mammography screening or not and recent cervical screening or not. SURVEYLOGISTIC procedures were used for bivariate analysis and also for multivariable models. Two models were developed for each outcome of interest; the first model included socio-demographic, SES and healthcare access variables, and the second included individual and country level covariates. Odds ratios and 95% confidence intervals were reported; and a p-value of less than 0.05 was considered statistically significant. List-wise deletion was used to deal with missing data. Data were expected to be missing at random since the WHS was a comprehensive survey designed to elicit responses to a wide range of questions, so there was likely no correlation between missing responses and our specific outcomes of interest.

## Results

The majority of the women in the analytic sample (women who had visited a healthcare facility in the past 12 months) were less than 40 years old (60%), married (71%), had only a primary school education or less (85%), were not currently working for pay (72%), and 79% of the women resided in rural areas (Table 1). During their last visit to a healthcare facility in the past year, most of the women had visited an NGO or other type of healthcare provider (36%), 35.7% had visited a government healthcare facility and 28% had visited a private healthcare facility. During the visit, 43% had seen a medical doctor, 22% had seen a nurse/midwife, while 36% had seen a traditional health provider. The majority of the women (82%) used public transportation to get to the facility, 15% walked or biked while 2% used a private vehicle. Travel time to the facility was less than 30 minutes for 67% of the women, between 30 minutes to 1 hour for 17%, and over 1 hour for 16%.

**Table 1:** Distribution of Study Characteristics, 2003 World Health Survey.

	Total^a^	Pelvic Exam/Pap Smear^b^		Mammography^c^	
	No (%)	No (%)	Crude OR (95% CI)	No (%)	Crude OR (95% CI)
**Age**					
>60	883 (8.2)	13 (1.4)	1 (Ref)	22 (18.9)	1 (Ref)
40–60	3126 (31.4)	184 (16.6)	3.2 (1.5–6.7)	118 (81.1)	0.9 (0.3–2.4)
<40	6964 (60.4)	842 (82.1)	8.5 (4.2–17.2)	-	-
**Marital Status**					
Married/Cohabiting	7512 (71.0)	829 (85.5)	1 (Ref)	87 (74.3)	1 (Ref)
Single	1336 (13.2)	103 (7.9)	0.5 (0.3–0.7)	2 (1.6)	0.7 (0.1–4.7)
Divorced/Separated/Widowed	2124 (15.8)	107 (6.5)	0.3 (0.2–0.5)	51 (24.0)	0.7 (0.4–1.2)
**Education**					
Primary school	9338 (85.3)	763 (70.7)	1 (Ref)	116 (84.6)	1 (Ref)
Secondary school	1357 (12.2)	217 (24.9)	2.6 (1.9–3.5)	14 (10.9)	3.2 (1.2–8.8)
College plus	267 (2.5)	56 (4.4)	2.2 (1.3–3.7)	10 (4.5)	2.9 (0.8–11.1)
**Employment**					
Government	293 (1.8)	56 (4.3)	1 (Ref)	10 (9.0)	1 (Ref)
Non-Govt/Self-Employed	4386 (26.3)	416 (39.6)	0.6 (0.4–0.9)	64 (29.4)	0.2 (0.1–0.6)
Not working for pay	6039 (71.9)	518 (56.2)	0.3 (0.2–0.5)	57 (61.4)	0.2 (0.1–0.4)
**Setting**					
Urban	3361 (20.9)	536 (40.8)	1 (Ref)	59 (50.9)	1 (Ref)
Rural	7612 (79.1)	503 (59.2)	0.4 (0.3–0.5)	81 (49.1)	0.2 (0.1–0.4)
**Household SES**					
High	3510 (35.3)	444 (52.6)	1 (Ref)	64 (66.6)	1 (Ref)
Middle	3425 (35.5)	271 (29.3)	0.5 (0.4–0.7)	29 (20.6)	0.3 (0.1–0.7)
Low	3259 (29.2)	183 (18.1)	0.4 (0.3–0.6)	24 (12.9)	0.2 (0.1–0.5)
**Last Facility Visited**					
Government	6044 (35.7)	584 (52.1)	1 (Ref)	62 (34.4)	1 (Ref)
Private	3092 (28.3)	315 (33.2)	0.8 (0.6–1.0)	57 (48.3)	1.5 (0.9–2.5)
NGO/Other	1612 (36.0)	101 (14.7)	0.3 (0.2–0.4)	15 (17.3)	0.4 (0.1–1.0)
**Last Provider Seen**					
Medical Doctor	4957 (42.5)	504 (50.9)	1 (Ref)	72 (69.6)	1 (Ref)
Nurse/Midwife	3849 (21.5)	423 (38.9)	1.5 (1.2–2.0)	50 (19.6)	0.7 (0.4–1.3)
Traditional/Other	2006 (35.9)	77 (10.2)	0.2 (0.2–0.3)	13 (10.8)	0.2 (0.1–0.4)

a Total: Women ages 18–64 years who have been to a healthcare facility in the past year.

b Pelvic Exam/Pap Smear: Women ages 18–69 years who have received a pelvic examination and/or pap smear in the past 3 years and has been to a healthcare facility in the past year.

c Mammography: Women ages 40–69 years who have received a mammography screening test in the past 5 years and has been to a healthcare facility in the past year.

95% CI: 95% Confidence Interval; Ref: Reference Group.

**Figure 1:**
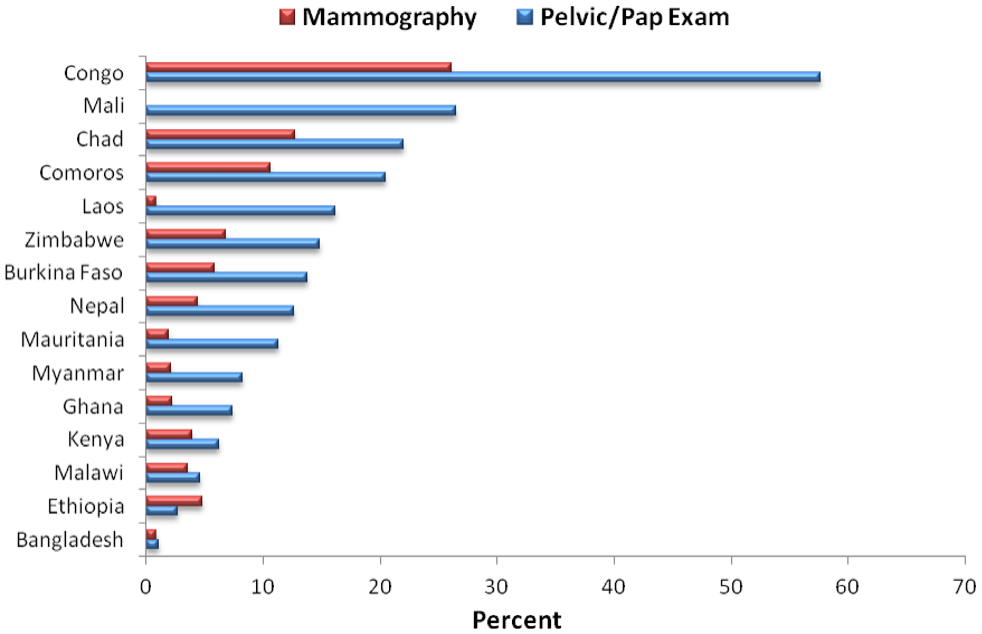
Pelvic Exam/Pap and Mammography Screening among Women Ages 18–69 (Pelvic/Pap) and 40–69 (Mammography) in Low-Income Countries, 2002–2003.

Overall, 4.1% of women ages 18–69 years in developing countries had received a pelvic exam or pap smear in the past three years. There were wide variations in the prevalence of cervical cancer screening by country, ranging from 1.1% in Bangladesh to 57.6% in Congo (Figure 1). Among women ages 40–69, 2.2% had received a mammography exam in the past 5 years, ranging from 0% in Mali to 26% in Congo.

Factors that significantly increased the likelihood of receiving a pelvic exam or pap smear in the past 3 years among 18 to 69 year old women included being younger than 60 years, having a secondary school education or greater, and seeing a nurse/midwife at the last health center visit (Table 1). Being single, divorced or widowed, not currently employed, residing in a rural area, residing in a low or middle SES household, visiting an NGO clinic, seeing a traditional health practitioner and having to walk or bike to the health center were variables significantly associated with reduced likelihood of a pelvic exam or pap smear. Among women ages 40 to 60 years old, having at least a secondary school education and a travel time of less than 30 minutes to the health center were associated with increased likelihood of receiving a mammography exam in the past 5 years. Being currently unemployed, residing in a rural, low or middle SES household, seeing a traditional health practitioner and having to walk or bike to the health center were significantly associated with reduced likelihood of a mammography exam.

In multivariate analysis of pelvic exam or pap smear among 18 to 69 year old women, after adjusting for age, education, employment and marital status, residing in the lowest SES household was associated with a 43% reduction (OR = 0.57, 0.39–0.82) and residing in the middle SES household was associated with a 33% reduction (OR = 0.66, 0.48–0.92) in the likelihood of receiving a pelvic exam/pap smear (Table 2). In addition, visiting an NGO instead of a government or private clinic (OR = 0.33, 0.23–0.49) and residing in a rural area (OR = 0.44, 0.32–0.60) were also associated with reduced likelihood of receiving a pelvic exam/pap smear. After adjusting for fixed effect of country covariates, these variables remained statistically significant and their effects appeared to be even stronger (Table 2). In addition, every unit increase in country health expenditure (as a percent of GDP), increased the likelihood of pelvic exam/pap smear by 50% (OR = 1.50, 1.30–1.73).

**Table 2:** Socio-Demographic Economic and Healthcare Access Determinants of Cervical Cancer Screening.

	Pelvic Exam/Pap Test in past 3 years among 18–69 year old women [Odds Ratio (95% CI)]	
Covariates	Model 1: SES and Healthcare Access^a^	Model 2: Plus Fixed Effects of Country Covariates^b^
**Household SES**		
Richest	1 (Ref)	1 (Ref)
Middle	0.66 (0.48–0.92)	0.49 (0.34–0.73)**
Poorest	0.57 (0.39–0.82)*	0.37 (0.23–0.58)***
**Travel Time**		
>1 hour	1 (Ref)	1 (Ref)
<30 minutes	0.84 (0.58–1.21)	0.72 (0.45–1.14)
30 minutes–1 hour	0.82 (0.54–1.26)	0.86 (0.51–1.44)
<30 minutes	0.84 (0.58–1.21)	0.72 (0.45–1.14)
**Travel Mode to Facility**		
Public	1 (Ref)	1 (Ref)
Private	0.79 (0.47–1.33)	0.76 (0.44–1.32)
Walked/Bicycle	0.64 (0.48–0.86)	0.70 (0.49–0.99)*
**Last Facility Visited**		
Government	1 (Ref)	1 (Ref)
Private	0.73 (0.55–0.98)	0.85 (0.57–1.25)
NGO/Other	0.33 (0.23–0.49)***	0.29 (0.17–0.52)***
**Setting**		
Urban	1 (Ref)	1 (Ref)
Rural	0.44 (0.32–0.60)***	0.44 (0.32–0.63)***
**Health Expenditure (% of GDP)**		1.50 (1.30–1.73)***
**Under-5 Mortality**		1.00 (0.99–1.00)
**GNI**		1.00 (1.00–1.01)**

a Model 1:adjusting for age, education, employment and marital status.

b Model 2:including country level covariates.

95% CI: 95% Confidence Interval; Ref: Reference Group.

In multivariate analysis of mammography screening among women ages 40 to 69, several two-way interaction terms were found to be significant predictors (Table 3). First, among women whose travel mode to a healthcare facility during their last visit was by public transportation, residing in a middle SES household compared with a high SES household was associated with reduced likelihood of mammography screening (OR = 0.14, 0.04–0.50). Secondly, among women residing in rural areas, belonging to a middle SES household compared with high SES was also associated with reduced likelihood of mammography screening (OR = 0.14, 0.04–0.50). Among women residing in urban areas, a similar but stronger reduction in the likelihood of screening was also observed (OR = 0.01, 0.00–0.07). Thirdly, among women residing in rural areas, a travel time of 30 minutes to 1 hour compared with over one hour of travel was associated with a significant increase in the likelihood of mammography screening (OR = 3.25, 1.09–9.75). in contrast, among women residing in urban areas, a travel time of 30 minutes to 1 hour compared with over an hour of travel reduced the likelihood of mammography screening (OR = 0.06, 0.00–0.72).

**Table 3:** Socio-Demographic Economic and Healthcare Access Determinants of Breast Cancer Screening.

		Mammography Test in Past 5 Years among 40–69 year old women [Odds Ratio (95% CI)]	
Covariates		Model 1: SES and Healthcare Access^a^	Model 2: Plus Fixed Effects of Country Covariates^b^
**Last Facility Visited**			
Government		1 (Ref)	1 (Ref)
Private		1.61 (0.95–2.71)	2.00 (1.12–3.59)
NGO/Other		1.03 (0.34–3.13)	0.70 (0.23–2.15)
Travel Mode = Public	Richest	1 (Ref)	1 (Ref)
	Middle	0.14 (0.04–0.50)	0.02 (0.00–0.15)
	Poorest	0.33 (0.08–1.31)	0.04 (0.00–0.31)
Travel Mode = Bike/Walking	Richest	1 (Ref)	1 (Ref)
	Middle	1.62 (0.50–5.24)	0.16 (0.04–0.69)
	Poorest	1.29 (0.44–3.80)	0.07 (0.02–0.29)
Setting = Rural	Richest	1 (Ref)	1 (Ref)
	Middle	0.14 (0.04–0.50)	0.11 (0.02–0.56)
	Poorest	0.33 (0.08–1.31)	0.25 (0.04–1.45)
Setting = Urban	Richest	1 (Ref)	1 (Ref)
	Middle	0.01 (0.00–0.07)	0.02 (0.00–0.15)
	Poorest	0.17 (0.02–1.28)	0.04 (0.00–0.30)
Setting = Rural	>1 hr	1 (Ref)	1 (Ref)
	30 mins–1 hr	3.25 (1.09–9.75)	0.67 (0.08–5.71)
	<30 mins	1.84 (0.81–4.19)	3.02 (0.46–19.86)
Setting = Urban	>1 hr	1 (Ref)	1 (Ref)
	30 mins–1 hr	0.06 (0.00–0.72)	2.52 (0.72–8.86)
	<30 mins	2.18 (0.24–19.98)	1.39 (0.61–3.18)
Health Expenditure (% GDP)			1.00 (0.75–1.35)
Under-5 Mortality			1.02 (1.00–1.03)
GNI			1.00 (0.99–1.00)

a Model 1:adjusting for age, education, employment and marital status.

b Model 2:including country level covariates.

95% CI: 95% Confidence Interval; Ref: Reference Group.

After adjusting for country level covariates including health expenditure (as a % of GDP), under-5 mortality rate and GNI, a significant association between type of healthcare facility and mammography screening was observed (Table 3). Compared with visiting a government run facility, private healthcare facilities appeared to increase the likelihood of mammography screening among women by twofold (OR = 2.00, 1.12–3.59). In addition, women residing in middle or low SES households appeared to have significantly lower likelihood of mammography screening regardless of residence in a rural or urban area as well as mode of transportation. The only country level covariate found to be a significant predictor of mammography screening was under-5 mortality rate; there was a 2% increase in the likelihood of receiving mammography for every unit increase in the under-5 mortality rate.

## Discussion

This study focused on assessing the rates and determinants of breast and cervical cancer screening among women residing in developing countries. In order to assess the influence of health care access on screening rates, this analysis was restricted to women who had visited a healthcare clinic for themselves or their children in the past year. Generally, screening rates were very low, although there were wide variations between countries. Mammography screening rates were very low in most countries, while cervical cancer screening by pelvic examination and/or Pap smear was more common. Other studies have reported these low screening rates and wide variation by country previously [26], [27]. The low rates of mammography screening in this population is not surprising given the extensive cost and advanced infrastructure required to have systemic mammography facilities and trained personnel. Many organizations such as the WHO do not recommend routine mammography screening for poor countries, focusing instead on Breast Self Examination which is a cheaper option [34].

Several factors influence the actual receipt of breast and cervical cancer screening in developing countries. The focus of this analysis was on socio-economic status and healthcare access determinants of screening. After adjusting for several important individual level covariates such as age, education, employment and martial status, belonging to the poorest SES household, living in a rural community and visiting an NGO clinic (versus a government or private run clinic) were significantly associated with lower likelihood of receiving cervical cancer screening. There were no significant associations between travel time and travel mode and cervical cancer screening. After adjusting for country level characteristics related to health expenditure, these same covariates remained significant. In addition, country expenditure on health (as % of GDP) had a very large impact on cervical cancer screening. This suggests that irrespective of individual and neighborhood factors, the government's investment in the health infrastructure has the potential for significantly improving cancer screening rates within a country. This aligns with other studies which have generally shown that increased health expenditure improves health outcomes [35], [36]. Such investment could influence screening rates through better equipments and trained personnel in hospitals, or better outreach and education of women about the importance of screening.

Although mammography screening rates were generally low, there were significant differences in the likelihood of obtaining a mammography test in the past 5 years based on SES, rural/urban residence and travel time. The only country level covariate that was significantly associated with mammography screening was the under-5 mortality rate. It is possible that for developing countries, the cost of having a functional mammography screening facility is so prohibitively high that within limited budgets, even major increases in health expenditure (as % of GDP) are inadequate. While unintuitive, under-5 mortality rate may increase the likelihood of receiving a mammography test through increased contact with the healthcare system. It is possible that a high under-5 mortality rate within the country may force women to have more frequent contact with hospitals and healthcare personnel who may also recommend health check-ups for the mothers including potentially cancer screening.

This study is the first of its kind to assess the contribution of SES, the health care system and country-level healthcare expenditure on cancer screening in developing countries. Important factors that were associated with cancer screening include SES; the poorer the household, the less likely it is to get screened. This association has also been reported previously in different populations [37]–[43]. This may be because low SES households tend to be less educated and less likely to be aware of the benefits of cancer screening or are likely to have negative perceptions about screening. It is also likely that working low-paying, menial jobs leaves little time or opportunity to participate in screening programs. This analysis shows that the influence of SES on breast cancer screening varies depending on rural/urban residence. For instance, in urban areas, women belonging to middle SES households have much lower odds of receiving a mammography test compared with women belonging to middle SES households in rural areas. This may be due to the lack of community resources, and the higher cost of living in urban areas compared with rural areas that provide less disposable income for urban middle SES households to be spent on healthcare.

A major strength of this study is the assessment of socio-economic and health system factors from developing countries surveyed as part of the 2003 World Health Survey. The standardized protocol and questionnaires allowed for the use of data from several developing countries. Also, the use of PCA to define household SES allowed for the inclusion of household assets and did not rely only on income and education. This improves the validity of the SES measure by accounting for differences in SES between rural and urban areas, and between countries. In addition, the availability of country level variables from the World Bank allowed for the assessment of the impact of country characteristics on cancer screening. Limitations of this analysis include the survey nature of the responses which may be vulnerable to recall bias on the part of the respondents. Also, the health system characteristics assessed in this study was based on the last healthcare facility attended in the past year. It is possible that this is not representative of the facility that a woman would normally attend for cancer screening.

In summary, cancer screening rates in developing countries are generally very low. Unfortunately, there are large projected increases in cancer rates in developing countries in the coming decades due to the increasing westernization of lifestyle as well as environmental factors. If we are to meet the challenge of the rising cancer epidemic in these regions of the world, it will be very important to understand the factors that inhibit and encourage cancer screening in order to promote early detection. Some of these barriers include factors related to healthcare access such as availability and accessibility of healthcare facilities and healthcare providers, as well as the lack of a comprehensive approach to cancer prevention, screening and treatment on a national level. In addition, some countries may require extra efforts aimed at including men in cancer education programs about the necessity for routine breast and cervical cancer screening for their women, since in many cases their permission may be needed.

Developing countries face monumental challenges; ranging from socio-economic to infrastructural and massive infectious disease epidemics. However, these challenges must not detract from the importance of understanding and addressing barriers to proper cancer screening. Based on this analysis, improving the quality and access to government run hospitals especially in rural areas and increasing the government expenditure on health (as % of GDP) appear to be ways by which cancer screening can be improved.
